# Temporary Memory Steal: Transient Global Amnesia Secondary to Nephrolithiasis

**DOI:** 10.5811/cpcem.2018.9.39338

**Published:** 2018-09-28

**Authors:** Muhammad Durrani, Jerry Milas, Gregory Parson, Richard Pescatore

**Affiliations:** Inspira Medical Center, Department of Emergency Medicine, Vineland, New Jersey

## Abstract

Transient global amnesia (TGA) is typified by an abrupt and transient anterograde amnesia, “with repetitive questioning and often variable retrograde amnesia persisting up to 24 hours.” A 54-year-old male presented to our emergency department with paroxysms of left-sided flank pain, suggestive of renal colic. Computed tomography (CT) of the abdomen/pelvis revealed a three-millimeter left ureterovesicular-junction calculus. Pain control proved difficult, necessitating multiple doses of opioid and non-opioid analgesia. Subsequently, the patient developed repetitive questioning and perseveration with anterograde amnesia with a negative CT brain and unremarkable further workup. He experienced a complete resolution of symptoms within a 24-hour period, with a discharge diagnosis of TGA secondary to nephrolithiasis. This is the third case of TGA attributed to nephrolithiasis in the medical literature.

## INTRODUCTION

Transient global amnesia (TGA) is an acute-onset clinical entity typified by an abrupt and transient anterograde amnesia, “with repetitive questioning and often variable retrograde amnesia persisting up to 24 hours.”^1^,^2^ Inherent to the diagnosis of TGA is the preservation of neurologic functioning including procedural memory (ability to remember and apply a series of steps to a task).^2^ Diagnostic criteria for TGA demand that there be “no clouding of consciousness, other impairments of cognition, or a history of epilepsy or head trauma.”^3^ Several studies have postulated that the mechanisms of TGA are comparable to processes underlying “cerebral ischemia, epilepsy, and migraines, or may arise from disturbance of venous hemodynamics.”^4^ Yet more than a century after it was first described, there is no definitive evidence supporting any of these mechanisms.

Recently, a growing body of evidence supports the role of emotional and psychological factors as precipitating events in up to 90% of reported TGA cases.^5^,^6^ Recent magnetic resonance imaging (MRI) data on individuals following an episode of TGA show development of small hippocampal lesions as a result of increased vulnerability of selective neurons from metabolic stress, further supporting the postulation that TGA is triggered by transitory “stress-induced inhibition of memory formation in the hippocampus.”^4^ TGA occurs in men and women equally with a mean age of 50–70 years. The alarming symptoms often culminate in an emergency department (ED) visit with the incidence ranging from 3–10 per 100,000 patients per year.^7^ No laboratory investigations or imaging modality can confirm the diagnosis of TGA. Thus, the diagnosis “relies on a detailed clinical history, cognitive evaluation, and physical examination.”^1^ The diagnosis is also dependent on eliminating other life-threatening etiologies including toxidromes, metabolic derangements, cerebrovascular accident (CVA), seizure activity, and central nervous system (CNS) infections.^8^ Although, there is no specific treatment for TGA, when alternative diagnoses are suspected, focused investigation, treatment, and secondary prevention should be pursued to address those clinical entities. TGA episodes are self-limited, and improvement is noted within 24 hours without any intervention with favorable short- and long-term prognosis.^1^

## CASE REPORT

A 54-year-old male with a past medical history of nephrolithiasis and hypertension arrived to our ED at 9:55 a.m. with complaints of left flank pain with nausea and vomiting. The patient noted that he had experienced similar paroxysms of pain with his previous episode of nephrolithiasis. He described his current symptoms as starting suddenly four hours prior to arrival to the ED. The pain was localized to his left flank with no alleviating or exacerbating factors, and he described it as a sharp sensation with radiation to the left inguinal region. The patient rated this pain numerically as a 10/10 in severity with associated nausea and episodes of non-bloody, non-bilious vomiting.

Upon arrival, his vitals revealed a blood pressure of 179/87 millimeters of mercury, pulse 63 beats per minute, respiratory rate 16 breaths per minute, 100% oxygen saturation on room air and temperature of 97.0°F. His blood glucose was 132 milligrams per deciliter (mg/dL). Lab work obtained included a complete blood count, complete metabolic panel, and urinalysis, which were found to be unexceptional aside from microscopic hematuria. The patient had a similarly unremarkable physical examination. A computed tomography (CT) of his abdomen and pelvis was obtained, and attempt at parenteral analgesia with intravenous (IV) ketorolac 15mg followed by hydromorphone 1mg IV were done. The CT revealed a three-millimeter obstructing calculus at the left ureterovesicle junction (UVJ) with left-sided perinephric stranding and mild hydronephrosis.

The patient continued to have significant pain despite continued opioid dosing. Shortly after his CT, the patient’s wife alerted ED staff that he was repeatedly asking her, “How did I get here?” and “Why am I here?” The patient was evaluated, and his physical examination was repeated to reveal no neurologic deficits. His presentation and physical examination were not consistent with opioid intoxication or medication side effect as he maintained his respiratory drive at baseline as well as his procedural memory, and was not opioid naïve, having received similar medications during his previous episode of renal colic. It was revealed at this point that the patient did not have any recall about his IV placement, lab draws, or his CT. The decision was made to obtain a non-contrast CT of his brain, which revealed no acute intracranial abnormalities. Because of the patient’s presentation and clinical course, the decision was made to admit him to the observation unit and obtain a neurology consult for possible TGA secondary to stress and pain induced by the patient’s nephrolithiasis. The neurologist consulted obtained a thorough history and examined the patient. He underwent MRI and magnetic resonance angiography (MRA) of the brain, electroencephalography (EEG), carotid ultrasound, and echocardiogram, as well as further lab testing that included a thyroid stimulating hormone, vitamin B-12 levels, folic acid level, and rapid plasma reagin testing. All additional testing was normal. The patient’s symptoms resolved within the same day of admission (within 24 hours). He was discharged with a diagnosis of TGA.

**CPC-EM Capsule**

What do we already know about this clinical entity?

Transient Global Amnesia (TGA) is characterized by transient anterograde amnesia with repetitive questioning, no focal deficits, and preservation of procedural memory, cognition, and identity.

What makes this presentation of disease reportable?

This is the third case-report of nephrolithiasis-related amnesia in the literature, where a painful experience induced sympathetic activation and precipitated TGA.

What is the major learning point?

TGA is commonly misdiagnosed, with diagnosis being primarily clinical and can be made if diagnostic criteria as described by Hodges and Warlow are fulfilled.

How might this improve emergency medicine practice?

An understanding of the precipitating factors, and diagnostic criteria can focus the investigation and differential diagnosis to more accurately diagnose TGA.

## DISCUSSION

TGA, with its abrupt development of prolific symptoms of dense anterograde amnesia, “remains one of the most enigmatic syndromes in neurology.”^1^ Although most literature on TGA comes from the neurology realm, it is a syndrome that is encountered by the full range of medical specialties, particularly emergency medicine. Although uncommon, it is paramount to be able to distinguish TGA from other life-threatening clinical entities because while the prognosis of TGA is generally benign, other similar disease states carry potential for life-threatening sequelae.^9^ The diagnosis of TGA is primarily clinical and can be made if diagnostic criteria, as described by Hodges and Warlow and adapted from Caplan, are fulfilled (Figure). These include the presence of an anterograde amnesia that is “witnessed by an observer, no clouding of consciousness or loss of personal identity, cognitive impairment limited to amnesia, no focal neurological or epileptic signs, no recent history of head trauma or seizures, and resolution of symptoms within 24 hours.”^8^ The hallmark of TGA is anterograde amnesia with freshly acquired memories at greatest risk, while long-term memories, self-awareness, and procedural memory, “as well as an awareness of what one should know, are typically preserved.”^10^ Repetitive questioning is frequently reported during an episode of TGA, likely due to an inability to acquire new information coupled with retrograde memory loss, with one case series showing 90% of patients experiencing repetitive questioning.^11^ Patients suffering from TGA will return to baseline within a few hours, except for a “dense, residual amnestic gap for events that occurred during the TGA attack.”^12^ Helpful in making the diagnosis of TGA is the occurrence of a stressful precipitating event. “Up to 90% of TGA episodes have an identifiable physical or psychological precipitating factor.”^13^

The differential diagnosis of TGA includes transient ischemic attack (TIA), subarachnoid hemorrhage, complex partial seizures, transient epileptic amnesia, psychogenic amnesia, drug-related states, metabolic derangements, CNS infections, and toxic ingestion. Misdiagnoses often occur in patients with TGA, as patients are often labeled without adherence to the criteria with diagnoses such as CVA, migraine headaches, and epileptic discharges. In the case presented, the episode was witnessed with noted anterograde amnesia and a clear distinct precipitating event. Additionally, the patient maintained his consciousness during the episode, with a normal neurological examination, and had resolution of symptoms within 24 hours. The patient underwent further testing with MRI, MRA, and EEG, but it should be noted that these studies are often normal in TGA and cannot be used to reliably diagnose the condition.

Studies that have examined the different precipitating factors contributing to the development of TGA have classically grouped these events (in order of prevalence) as physical effort, emotional stress, temperature changes, other factors, post-coital, and lastly acute pain. Nephrolithiasis as an inciting cause of TGA, especially in an individual with prior episodes of renal colic, has only been described twice previously and should reinforce the varied presentation by which TGA may manifest.^13^ A search of MEDLINE, PubMed, and Scopus revealed only two cases of TGA attributed to nephrolithiasis.^14^,^15^ Our case is the third such presentation of TGA attributed to nephrolithiasis, where a severe, painful experience induced sympathetic activation and precipitated TGA.^14^ As the prognosis of TGA is extremely favorable, an important aspect of management after diagnosis is meeting the psychological needs of the patient and his or her family.

## CONCLUSION

In summary, the patient’s episode of TGA was precipitated by the acute stress induced by his nephrolithiasis. This article is the third case report of nephrolithiasis-related amnesia in the medical literature. This case should serve to reinforce the unique presentation of TGA and the different factors that may precipitate an event, as well as the differential diagnosis that the emergency physician must consider to arrive at the diagnosis. TGA is commonly misdiagnosed, without adherence to diagnostic criteria, as TIA, complex migraine, and seizures. These misdiagnoses can result in unnecessary in-hospital workup, lifelong stigmata and changes in individual’s daily functioning, along with unwarranted, subsequent follow-ups and costly medical care, as there is no increased risk of TIA or increased mortality for patients with TGA.

Documented patient informed consent and/or Institutional Review Board approval has been obtained and filed for publication of this case report.

## Figures and Tables

**Figure:**
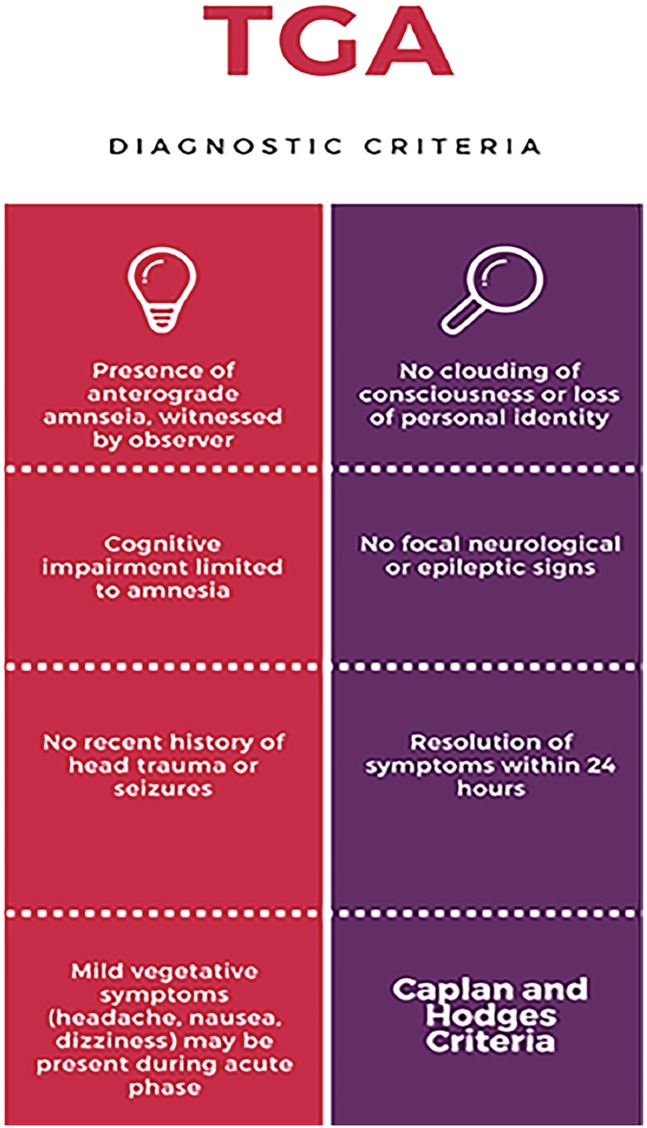
Diagnostic criteria for transient global amnesia.^3^
